# Mineral Content and Biochemical Variables of *Aloe vera* L. under Salt Stress

**DOI:** 10.1371/journal.pone.0094870

**Published:** 2014-04-15

**Authors:** Bernardo Murillo-Amador, Miguel Víctor Córdoba-Matson, Jorge Arnoldo Villegas-Espinoza, Luis Guillermo Hernández-Montiel, Enrique Troyo-Diéguez, José Luis García-Hernández

**Affiliations:** 1 Centro de Investigaciones Biológicas del Noroeste, S.C. La Paz, Baja California Sur, México; 2 Universidad Autónoma de Baja California Sur, La Paz, Baja California Sur, México; 3 Universidad Juárez del Estado de Durango, Durango, Durango, México; University of Heidelberg, Germany

## Abstract

Despite the proven economic importance of *Aloe vera*, studies of saline stress and its effects on the biochemistry and mineral content in tissues of this plant are scarce. The objective of this study was to grow Aloe under NaCl stress of 0, 30, 60, 90 and 120 mM and compare: (1) proline, total protein, and enzyme phosphoenolpyruvate carboxylase (PEP-case) in chlorenchyma and parenchyma tissues, and (2) ion content (Na, K, Ca, Mg, Cl, Fe, P. N, Zn, B, Mn, and Cu) in roots, stems, leaves and sprouts. Proline and PEP-case increased as salinity increased in both parenchyma and chlorenchyma, while total protein increased in parenchyma and decreased in chlorenchyma, although at similar salt concentrations total protein was always higher in chlorenchyma. As salinity increased Na and Cl ions increased in roots, stems, leaves, while K decreased only significantly in sprouts. Salinity increases typically caused mineral content in tissue to decrease, or not change significantly. In roots, as salinity increased Mg decreased, while all other minerals failed to show a specific trend. In stems, the mineral concentrations that changed were Fe and P which increased with salinity while Cu decreased. In leaves, Mg, Mn, N, and B decreased with salinity, while Cu increased. In sprouts, the minerals that decreased with increasing salinity were Mg, Mn, and Cu. Zinc did not exhibit a trend in any of the tissues. The increase in protein, proline and PEP-case activity, as well as the absorption and accumulation of cations under moderate NaCl stress caused osmotic adjustment which kept the plant healthy. These results suggest that Aloe may be a viable crop for soil irrigated with hard water or affected by salinity at least at concentrations used in the present study.

## Introduction

Sodic lands are increasing worldwide and are creating challenges especially for developing countries in order to meet productivity needs with increasing population growth [1]. Salt stress is known to be a limiting factor in plant growth and yield [2] and is presently impairing at least 20% of crop productivity worldwide [3]. Saline environments can cause a wide number of responses in plants, including readjustment of transport and metabolic processes that can lead to growth inhibition [4]. The negative effects of salinity on plant growth are associated with low osmotic potential, nutritional imbalance, specific ion effect, or a combination of these factors in the soil [5].

In order to mitigate effects and find uses for low or unproductive lands, salt tolerant accessions are being identified, as well as new sodic tolerant industrial crops. Some of these crops studied with respect to salt tolerance and its effects include *Lesquerella fendleri* (Gray) S. Wats, a relatively new crop with seed oil that contains lesquerolic acid which is useful in the production of a biological lubricant, where it was found that a single cycle of selection *L. fendleri* in salinized conditions resulted in an improved line [6]; maize (*Zea mays* L.), where varieties tolerant to salt was studied and one variety showed to be more tolerant to salt stress than another [7]; *Coriandrum sativum* L., a herb, where the essential oil yield increased significantly with low NaCl and decreased significantly under high salinity [8]; *Mentha pulegium* L., a medicinal herb where the essential oil yield was found to be 2.75 higher in salt-treated plants. *Aloe vera* L. is no exception and is being considered as an alternative crop for industrial applications in sodic lands due to its high market demand and export potential [1]. In addition, it is known that *Aloe vera* grows well on saline soils of beach areas worldwide in tropical and subtropical xerophytic conditions [1].

In Aloe many studies have been carried out evaluating parameters such as growth, biomass, tissue water level, gel and aloin content under different geoecological conditions, such as pH and irradiance intensities. A few recent studies are those of [9] and [10] evaluated growth, biomass, ions, as well as water, gel and aloin content. More recently, Zapata *et al*. [11] evaluated the gel from different Aloe species as an antifungal treatment. In *Aloe vera* the innermost part of the leaf is clear, soft, moist and is a slippery tissue that consists of large thin-walled parenchyma cells in which water is held in the form of a viscous mucilage [12], while the shallowest part of the leaf is called the chlorenchyma, which contain the main photosynthetic cells of the plant and manufacture carbohydrates during photosynthesis and form the basic green tissue of plant leaves. The influence of salinity on plant cell ultrastructure, physiology and expression of genes is well documented, but the influence of salt stress on these tissues is not fully understood. Information about the influence of salinity on the properties of these individual tissues in Aloe plants is still lacking.

In contrast, to the aforementioned studies, detailed studies of the degree of salt tolerance of *Aloe vera* and its effects on plant productivity for use in optimized industrial application are incomplete. In plants in general, it is known that osmotic stress and ion-induced injury occur as a result of soil salinity [13], [14]. In this regard, Zheng *et al*. [15] for example, studied the plant growth and ionic distribution in relation to osmosis in *Aloe vera* at different salinity levels. Or more recently, Sahu *et al*. [16] reported growth, biomass, gel and aloin contents in two Aloe species *Aloe ferox* and *Aloe vera* with saline stress at different pH levels. Salt stress in combination with high pH apparently induces greater injury because the high pH environment surrounding roots may cause metal ions and phosphorus to precipitate [17], affecting absorption of minerals in the form of inorganic anions such as NO3^−^, H_2_PO_4_^−^, K^+^, Na^+^ and Cl^−^, disrupting ionic balance and pH homeostasis in tissues [18]. Thus, plants saline and alkaline soils must work doubly hard in order to maintain intracellular ionic balance from both NaCl induced osmotic imbalance as well ion toxicity in order to survive.

How well *Aloe* spp. performs in terms of absorption of minerals in saline soil conditions is unknown. There is one study that has evaluated sodicity levels in terms of growth, gel and nutrient concentration uptake, however they used units of ESP (exchangeable sodium percentage) which varies since is calculated by dividing exchangeable sodium (cmol/kg) by the total sum of all cation concentrations (cmol/kg) and then multiplying by 100. Without knowing the total cation concentration it is not possible to isolate effect of sodium alone. Different NaCl concentration or sea water have been used to evaluate salt tolerance in *Aloe vera* plants such as 100 and 200 mM NaCl [19], [20], 200 mM [21], 300 mM [22], including 60% sea water during 1.5 years [9], 100% sea water for 3.5 years [23] and even saline lake water EC: 3, 6, 9, 12, 15, 18, or 21 dS m^−1^ [24]. However, very little is known about *Aloe vera* tolerance and performance under low NaCl stress especially its effects on mineral and biochemical content in plant tissues. In this research, *Aloe vera* was studied for its response to low-salt treatments (NaCl), while pH was kept constant 6.5 to avoid secondary effects. The objective of this study was to address two specific issues regarding tissues of *Aloe vera* plants when grown under moderate saline conditions:1) does NaCl in the growth medium alter proline, total protein, and PEP-case content in different tissues and to determine if these could function as biochemical markers that could be associated with salt tolerance 2) does NaCl in the growth medium alter mineral/ion content such as Na, K, Ca, Mg, Cl, Fe, P. N, Zn, B, Mn, and Cu in different tissues and to determine if these inorganic ions could be related to salt tolerance.

## Materials and Methods

### 2.1 Study area

The experiment was conducted under greenhouse conditions in La Paz, located in a semiarid zone of Baja California Sur, northwest of Mexico (24°08′ 09.73″ N, 110°25′ 41.73″ W), 7 m above sea level. Mean, maximum and minimum temperatures in the greenhouse were 19.2, 35.3 and 5.3°C with 60% relative humidity. The meteorological observations were obtained during the study from an automated weather station located inside the greenhouse. The experimental site has a Bw (h´) hw (e) climate which is considered to be a semiarid climate and sustains xerophitic vegetation. The soils are characterized by good conditions of aeration and penetrability for plant roots and low-medium water retention, with high sand content, low organic matter content (less than 1%), neutral pH at the surface and are slightly alkaline at depths of 20–60 cm.

### 2.2 Plant material

In this study local wild ecotype of *Aloe vera* L. were obtained from the experimental field at the Centro de Investigaciones Biológicas del Noroeste, S.C. (CIBNOR). For locations/activities, no specific permissions were required and the field studies did not involve endangered or protected species. The plants were uniform in size, health, and color with a height of 30 cm. These plants were transplanted and cultured in circular plastic pots that contained a mixture of sand and peat-moss (1∶1, v:v) (Sunshine, Sun Gro Horticulture Canada, Ltd.) (One plant per pot) that were 25 cm high with a 20 cm diameter width at the upper surface and holes at the bottom for drainage. The pots were placed in a greenhouse and cultured with natural light. After being transplanted, 1 L of tap water was applied every three days during 51 days which was the time needed to attain total plant rooting. For the treatments with NaCl only uniform plants were selected.

### 2.3 Biochemical variables

Biochemical variables were measured in two tissues of Aloe leaves, chlorenchyma and parenchyma. Chlorenchyma is the green rind or cuticle of the *Aloe vera* plant that consist of multiple layers interspersed with chloroplasts, while parenchyma is the inner leaf pulp composed of larger thin-walled parenchyma cells that store the *Aloe vera* gel.

### 2.4 Total protein

For the extraction and determination of proteins, 2 g of tissue were frozen in liquid N_2_ and ground in a coffee grinder. After grinding, the fine powder was transferred to a 30-mL Corex tube, 10 mL of extraction buffer (100 mM monobasic potassium phosphate, 1% polyvinylpyrrolidone-40 (PVP-40) 2 mMEDTA, pH 7.0) was added and then shaken vigorously for 30 s. Once the tissue was homogenized, the mix was centrifuged at 8.645 *g* for 12 min at 4°C in an SS-34 rotor of a centrifuge. The supernatant was removed and aliquoted in Eppendorf tubes and stored at −20°C until use. Total proteins were quantified by the Bradford method [25].

### 2.5 Proline

Free proline content was estimated by following the method of Bates *et al*. [26]. Fresh 0.5 g of chlorenchyma and parenchyma samples was homogenized in 5 mL of 3% sulphosalicylic acid using a mortar and pestle. About 2 mL of extract was taken in test tube and to it 2 mL of glacial acetic acid and 2 mL of ninhydrin reagent were added. The reaction mixture was boiled in a water bath at 100°C for 30 min. After cooling the reaction mixture, 4 mL of toluene was added. After thorough mixing, the chromosphere containing toluene was separated and absorbance of red color developed was read at 520 nm against toluene black on UV-visible spectrophotometer. The proline concentration was determined using calibration curve and expressed as mg proline per g fresh weight of tissue.

### 2.6 Phosphoenolpyruvate carboxylase

Activity of the enzyme phosphoenolpyruvate carboxylase (PEP-Case) was determined spectrophotometrically at 340 nm by coupling the reaction to the oxidation of NADH in the presence of malate dehydrogenase (MDH). The standard assay medium contained the enzyme extract, 10 units of MDH, 0.1 mM NADH, 2.5 mM MgSO_3_ and 5 mM NaHCO in a total volume of 2.95 mL 50 mM Tricine buffer (pH 8.8). The reaction was started by the addition of 50 mL of PEP at 2.2 mM final concentration. The rate of oxidation of NADH was measured every 15 sec after the addition of PEP for a total time of 3 min. The reaction was observed using the visual display of the spectrophotometer to confirm the adequate mixing of the cuvette contents and that NADH oxidation reaction was linear. Assays were done in triplicate.

### 2.7 Mineral content of roots, stems, leaves and sprouts

The mineral content was measured in roots, stems, leaves and sprouts dried tissue. Mineral content in roots and stems were measured after plants were harvested at the end of the experiment. The roots and stems were rinsed with tap water until all particles of peat moss-sand were removed and then with distilled water. Mineral content of leaves were measured during the experimental period in those mature leaves (approximately 20–30 cm of length) that were cut at the base by knife sharp without damage the main plant. Each tissue of the main plant (roots, stems, and leaves) and sprouts were harvested and placed in a pre-heated oven (Shell-Lab, model Fx-5, serie-1000203), at 80 °C, until constant weight, in order to obtain the dry weight (g). Mineral content of sprouts was measured in those sprouts originating from the base of the main plant which were harvested and registered. After harvested, all leaves and sprouts (sprouts included roots, stems and leaves) were rinsed by dipping three times for a few seconds in distilled-deionized water. For mineral analysis, dry plant material was ground in a blender (Braun 4-041 Model KSM-2). The Na, Ca, Mg, Mn, Fe, Cu, Zn, and K content was determined by atomic absorption spectrophotometer (Shimadzu AA-660, Shimadzu, Kyoto, Japan) after digestion with H_2_SO_4_, HNO_3_, and HClO_4_ (1∶10∶4). Chloride was extracted in boiling water and determined by ion chromatography (Shimadzu HIC-6A, Shimadzu, Kyoto, Japan). Phosphorous was estimated colorimetrically as phosphomolybdate blue complex method at 660 nm from the same extract. Total nitrogen was determined by Kjeldahl digestion using a sulphuric acid and salicylic acid mixture with cupper and potassium sulphate such as catalysts followed by ammonium estimation using the Nessler colorimetric method. Boron was estimated through spectrophotometric method based on colorimetric technique with previous digestion using chlorhydric acid and curcumin in the presence of oxalic acid.

### 2.8 Experimental design

The saline treatments were 0, 30, 60, 90 and 120 mM de NaCl. The experiment was established under a completely randomized design with four replicates and each replicate consisted of three pots with one plant per pot. The saline treatments were applied after 51 days of tap water irrigation. All plants were watered daily with an excess of appropriate saline solution with nutrients containing (mg/L) 220 N, 40 P, 200 K, 140 Ca, 42 Mg, 4 Fe, 1.25 Mn, 0.18 B, 0.23 Zn and 0.25 Cu. Watered with an excess of solution (500 mL) permitted to flush the pots allowing draining the excess of solution to maintain the level of salinity. All drained solutions were collected to measure the electrical conductivity and verify that the salinity of the treatment solutions and the drained solutions were similar. The pH of all treatments solutions was maintained close to 6.5 by adding H_2_SO_4_ or KOH. Plants were grown in an average of 12-h photoperiod of 279±23-µmol m^−2^ s^−1^ photosynthetically active radiation. The length of the experiment was 150 days.

### 2.9 Statistical analysis

Biochemical variables proline, protein and PEP-case were analyzed using a two-factor MANOVA for two classifications of independent variables, the first being saline treatments (NaCl) and in tissues of the chlorenchyma and parenchyma. For mineral content the data was analyzed using a one way ANOVA with saline treatments (NaCl) as the independent variable. Analysis of variance and significant differences among means were determined and least significant differences were calculated using Tukeýs HSD test. In all cases, study factors were varied to provide a completely randomized design and differences among means were considered significant at *P*≤0.05. All analyses were done with Statistica software program v. 10.0 for Windows.

## Results

The MANOVA analysis for biochemical traits showed significant differences between NaCl treatments (Wilks = 0.0080, F = 32.20, *p* = 0.000001), tissues (Wilks = 0.015, F = 583.95, *p* = 0.000001), and the interaction of NaCl×tissue (Wilks = 0.031, F = 16.73, *p* = 0.000001) including all biochemical variables. The MANOVA analysis for mineral content showed significant differences between NaCl treatments (Wilks = 0.000004, F = 2.44, *p* = 0.02) including all variables measured. It can be seen that the relationship of Wilks possibilities is significant at the level of 0.01. This reaffirms that there are differences between the factors in this study in some of the measured variables, and strengthens the likelihood that the differences observed in the univariate analysis (ANOVA) performed on the variables, are real differences and not false positives or differences that occur simply by randomized chance [27].

### 3.1 Total protein

Significant differences between NaCl treatments (F_4,30_ = 15.34; *p* = 0.000001), tissues (F_1,30_ = 24.01; *p* = 0.00003) and the interaction of NaCl×tissues (F_4,30_ = 54.69; *p* = 0.000001) were observed for total protein content. In chlorenchyma, total protein was higher for control (0 mM) or at low salt stress (30 mM) and decreased as salinity increased; the contrary response was exhibited in parenchyma, where the higher values of total protein were found at 120 mM and decreased as salinity decreased. The sum of total protein was slightly higher in chlorenchyma than in parenchyma (Table 1).

**Table 1:** Mean fresh weight values of the interaction of NaCl×tissue of total protein, proline, and PEP-case activity of *Aloe vera* L. plants subjected to NaCl (mM) stress.

	Total protein (mg g^−1^)		Proline (mg g^−1^)		PEP-case (µmol NADH/g prot*min)	
NaCl	Chlorenchyma	Parenchyma	Chlorenchyma	Parenchyma	Chlorenchyma	Parenchyma
0	146.70 a	96.92 c	3.33 d	1.54 d	0.296 c	0.260 a
30	139.05 ab	121.80 b	3.95 c	2.49 c	0.299 c	0.265 a
60	134.58 b	129.39 ab	4.03 c	2.63 bc	0.318 bc	0.268 a
90	131.31 b	136.61 a	5.19 b	2.89 ab	0.341 b	0.273 a
120	113.23 c	140.64 a	5.63 a	3.06 a	0.416 a	0.279 a

Values within the same columns with same letters are not significantly different at *P*≤0.05 (Tukeýs HSD multiple range test).

### 3.2 Proline

Significant differences between NaCl treatments (F_4,30_ = 161.53; *p* = 0.000001), tissues (F_1,30_ = 1310.87; *p* = 0.000001) and the interaction of NaCl×tissues (F_4,40_ = 19.53; *p* = 0.000001) were observed in proline content. High content of proline was found in chlorenchyma than in parenchyma. In both tissues, proline content was lower at control and increased as salinity increased being higher at 120 mM (Table 1).

### 3.3 PEP-case

Significant differences between NaCl treatments (F_4,30_ = 18.92; *p* = 0.000001), tissues (F_1,30_ = 14.99; *p* = 0.000001) and the interaction of NaCl×tissues (F_4,30_ = 141.62; *p* = 0.000001) were observed for PEP-case activity. Higher values of PEP-case were found in chlorenchyma than in parenchyma. In chlorenchyma, the lower values of PEP-case were found at 0 mM and this content increased as salinity increased. In parenchyma, although not significant differences between NaCl were found, lower PEP-case was found at 0 mM and this content increased as salinity increased (Table 1).

### 3.4 Mineral content in roots

Salinity affected some minerals in roots. Significant differences between NaCl treatments were observed for K (F_4,15_ = 25.83; *p* = 0.000001), Cl (F_4,15_ = 141.62; *p* = 0.000001), N (F_4,15_ = 3.18; *p* = 0.04), and B (F_4,15_ = 11.41; *p* = 0.0001), while Ca (F_4,15_ = 1.61; *p* = 0.22), Mg (F_4,15_ = 2.95; *p* = 0.06), Na (F_4,15_ = 0.81; *p* = 0.53), Fe (F_4,15_ = 2.51; *p* = 0.08), P (F_4,15_ = 0.80; *p* = 0.53), Zn (F_4,15_ = 1.51; *p* = 0.24), Mn (F_4,15_ = 1.19; *p* = 0.35) and Cu (F_4,15_ = 1.20; *p* = 0.34) did not show significant differences. For those mineral with significant differences, the results show that K exhibited higher values at 0 mM and the lower at 60 mM. Chlorine content was higher at 120 mM and decreased as salinity decreased. Nitrogen was higher at 0 mM and lower at 30 mM, while B was higher at 120 mM and lower at 60 and 90 mM. Although calcium content was unaffected by NaCl, higher values of this ion was found at 90 and 120 and lower at 60 mM. Magnesium had higher values at 0 mM and decreased as NaCl increased. The control treatment had the lowest Na content and this ion increased as salinity increased. Iron was lower at 60 mM and higher at 90 mM and did not show a monotonic increasing or decreasing trend with NaCl. Phosphorus content was lowest at 60 mM and the control had the highest values. Zinc had lower values at any NaCl concentration and highest at 0 mM NaCl. Higher values of Mn were observed at 120 and 90 mM, while lower values were at 60 mM. The Cu content was higher values at 0 mM, and decreased at 30, 60 and 120 mM but increased newly at 90 mM. The K/Na ratio showed significant differences between NaCl treatments (F_4,15_ = 39.76; *p* = 0.000001) and decreased significantly as salinity increased, the contrary occurred for Na/K ratio, which increased as salinity increased and presented significant differences between NaCl treatments (F_4,15_ = 33.40; *p* = 0.000001) (Table 2).

**Table 2:** Mean values of dry weight (mg g^−1^) of the effect of NaCl (mM) in the mineral content of roots, stems, leaves and sprouts of *Aloe vera* L. plants subjected to saline stress.

Tissues	NaCl	Na/K	K/Na	Ca	Mg	K	Na	Cl	Fe	P	N	Zn	B	Mn	Cu
	0	0.90 c	1.11 a	17.75 a	5.83 a	24.94 a	22.57 a	7.41 c	0.64 a	3.47 a	14.50 a	0.061 a	0.10 a	0.041 a	0.021 a
	30	1.78 b	0.56 b	18.19 a	5.07 a	12.70 b	22.66 a	8.74 c	0.72 a	2.93 a	12.67 b	0.040 a	0.10 a	0.035 a	0.019 a
Roots	60	1.98 b	0.51 b	16.50 a	4.79 a	12.36 b	24.47 a	9.13 bc	0.58 a	2.88 a	13.00 ab	0.037 a	0.06 b	0.032 a	0.016 a
	90	2.62 a	0.38 b	19.82 a	4.39 a	9.53 b	25.00 a	11.90 ab	1.09 a	3.03 a	13.81 ab	0.046 a	0.06 b	0.042 a	0.020 a
	120	2.67 a	0.37 b	18.72 a	3.85 a	9.42 b	25.19 a	13.99 a	1.01 a	3.16 a	13.43 ab	0.038 a	0.11 a	0.045 a	0.018 a
	0	0.45 b	2.27 a	16.80 a	6.95 a	43.55 a	20.04 b	7.73 c	0.27 a	8.11 a	21.36 a	0.038 a	0.09 a	0.053 a	0.028 a
	30	0.84 b	1.18 b	19.83 a	7.57 a	28.15 b	23.58 ab	8.59 bc	0.30 a	8.56 a	21.33 a	0.032 a	0.07 a	0.044 a	0.025 ab
Stems	60	1.43 a	0.71 b	17.24 a	6.28 a	17.14 c	23.81 ab	8.89 bc	0.32 a	9.03 a	21.00 a	0.036 a	0.07 a	0.039 a	0.024 ab
	90	1.63 a	0.61 b	19.88 a	6.27 a	17.00 c	27.20 ab	12.44 ab	0.33 a	9.41 a	20.69 a	0.039 a	0.10 a	0.033 a	0.016 b
	120	1.82 a	0.56 b	18.15 a	6.92 a	15.34 c	27.80 a	14.57 a	0.39 a	9.46 a	22.43 a	0.038 a	0.08 a	0.051 a	0.016 b
	0	0.43 c	2.33 a	24.63 a	9.91 a	58.18 a	25.40 c	10.03 c	0.25 a	4.63 a	22.07 a	0.024 ab	0.12 a	0.11 a	0.028 a
	30	0.81 c	1.23 b	23.04 ab	8.62 ab	39.03 b	31.80 b	10.46 bc	0.23 a	4.60 a	16.99 b	0.020 b	0.11 ab	0.09 ab	0.030 a
Leaves	60	1.09 b	0.91 bc	19.90 bc	7.69 bc	31.71 c	34.58 ab	13.96 ab	0.23 a	4.16 a	15.58 bc	0.023 ab	0.11 ab	0.09 ab	0.032 a
	90	1.22 ab	0.81 bc	19.86 bc	6.46 c	30.27 c	35.28 ab	14.10 a	0.22 a	4.43 a	14.17 bc	0.021 b	0.10 b	0.06 b	0.034 a
	120	1.34 a	0.74 c	17.21 c	6.43 c	26.35 c	36.94 a	16.94 a	0.20 a	3.77 a	13.94 c	0.028 a	0.05 c	0.06 b	0.036 a
	0	0.24 d	4.10 a	15.88 a	7.10 a	75.51 a	18.43 c	9.78 c	0.33 a	6.72 a	21.82 a	0.038 a	0.08 b	0.05 a	0.045 a
	30	0.71 c	1.41 b	13.13 b	4.84 b	50.67 b	35.96 b	13.60 c	0.27 ab	6.23 a	17.69 b	0.037 a	0.13 a	0.019 a	0.043 a
Sprouts	60	1.07 b	0.94 bc	11.45 bc	3.81 c	42.32 c	45.12 a	19.09 b	0.26 ab	6.95 a	17.22 b	0.030 a	0.12 a	0.025 a	0.038 ab
	90	1.29 b	0.77 c	9.92 c	3.17 c	38.78 c	50.20 a	24.17 b	0.25 ab	6.41 a	17.10 b	0.031 a	0.06 b	0.016 a	0.035 b
	120	1.73 a	0.58 c	5.08 d	3.12 c	29.54 d	51.02 a	37.07 a	0.24 b	6.47 a	21.89 a	0.035 a	0.06 b	0.006 a	0.035 b

Values within the same columns with same letters are not significantly different at *P*≤0.05 (Tukey's HSD multiple range test).

### 3.5 Mineral content in stems

Significant differences between NaCl treatments were observed for K (F_4,15_ = 39.23; *p* = 0.000001), Na (F_4,15_ = 3.22; *p* = 0.04), Cl (F_4,15_ = 8.46; *p* = 0.0008), and Cu (F_4,15_ = 6.02; *p* = 0.004), while Ca (F_4,15_ = 2.30; *p* = 0.10), Mg (F_4,15_ = 0.90; *p* = 0.48), Fe (F_4,15_ = 0.74; *p* = 0.57), P (F_4,15_ = 0.85; *p* = 0.51), N (F_4,15_ = 0.70; *p* = 0.59), Zn (F_4,15_ = 0.45; *p* = 0.76), B (F_4,15_ = 2.35; *p* = 0.10), and Mn (F_4,15_ = 1.34; *p* = 0.29) did not present significant differences. For those mineral with significant differences, the results showed that K exhibited higher values at 0 mM and decreased as salinity increased. Sodium was affected by NaCl showing lower values 0 mM and this content increased as salinity increased. The same trend was observed for Cl, showing lower values at control and higher values as NaCl levels increased. The contrary was showed by Cu content which decreased as salinity increased. Calcium showed higher values at 30, 90 and 120 mM and lower values at 0 mM. Magnesium showed higher values under moderate salt stress (30 mM), decreased at 60 and 90 but increased at 120 mM without trend to NaCl. Iron content was lower at 0 mM and increased as salinity increased. Phosphorus showed higher values at 120 mM and decreased as salinity decreased. Nitrogen was higher at 120 mM followed by 0, 30, 60 and 90 mM. Zinc did not present a continuous monotonic trend with respect to NaCl, because it presented higher values at 90 mM, similar values at 120 and 0 mM and lower values at 30 and 60 mM. Boron was higher at 90 mM and lower at 30 and 60 mM. Manganese had higher values at 0 mM and decreased at the different NaCl levels. The K/Na ratio presented significant differences between NaCl treatments (F_4,15_ = 25.40; *p* = 0.000001) and was significantly lower as salinity increased. The Na/K ratio showed significant differences between NaCl treatments (F_4,15_ = 27.12; *p* = 0.000001) and increased as NaCl concentration increased (Table 2).

### 3.6 Mineral content in leaves

The results showed that salinity stress affected the majority of ions related to leaves, showing significant differences between NaCl treatments in Ca (F_4,15_ = 8.82; *p* = 0.0007), Mg (F_4,15_ = 12.31; *p* = 0.0001), K (F_4,15_ = 102.02; *p* = 0.000001), Na (F_4,15_ = 18.12; *p* = 0.00001), Cl (F_4,15_ = 12.11; *p* = 0.0001), N (F_4,15_ = 24.28; *p* = 0.000002), Zn (F_4,15_ = 5.10; *p* = 0.008), B (F_4,15_ = 24.87; *p* = 0.000002), and Mn (F_4,15_ = 8.59; *p* = 0.0008) content, while Fe (F_4,15_ = 2.08; *p* = 0.13), P (F_4,15_ = 1.72; *p* = 0.19), and Cu (F_4,15_ = 1.73; *p* = 0.19) do not showed significant differences. From those ions that showed significant differences, Ca, Mg, K, N, B, and Mn showed the higher values at 0 mM and decreased as salinity increased, while, Na, Cl and Cu showed the contrary, i.e., increased as salinity increased. Even if Zn showed higher values at 120 mM followed by 0, 60 and 30 mM, the lower values were showed at 90 mM. From those ions that didńt show significant differences, both minerals, Fe and P, showed higher values at 0 mM and decreased as salinity increased. The K/Na ratio showed significant differences between NaCl treatments (F_4,15_ = 47.96; *p* = 0.000001) and was reduced significantly as salinity increased (Table 2). Also, the Na/K ratio showed significant differences between NaCl concentrations (F_4,15_ = 72.61; *p* = 0.000001) and increased as NaCl concentration increased (Table 2).

### 3.7 Mineral content in sprouts

The results indicate that salinity stress affected the majority of ions related to sprouts, showing significant differences between NaCl treatments in Ca (F_4,15_ = 53.34; *p* = 0.000001), Mg (F_4,15_ = 93.22; *p* = 0.000001), K (F_4,15_ = 251.90; *p* = 0.000001), Na (F_4,15_ = 93.23; *p* = 0.000001), Cl (F_4,15_ = 77.88; *p* = 0.000001), Fe (F_4,15_ = 3.45; *p* = 0.03), N (F_4,15_ = 19.03; *p* = 0.00001), B (F_4,15_ = 42.51; *p* = 0.000001), Mn (F_4,15_ = 18.00; *p* = 0.00001) and Cu (F_4,15_ = 7.37; *p* = 0.001) content, while P (F_4,15_ = 2.19; *p* = 0.11) and Zn (F_4,15_ = 1.34; *p* = 0.30) did not show significant differences. From those ions that showed significant differences, Ca, Mg, K, Fe, Mn and Cu content was highest at 0 mM and decreased as salinity increased, while, Na and Cl trended inversely, i.e., increased as salinity increased. Even if N showed higher values at 120 followed by 0 mM, the lower values were found at 90 mM. From those ions that did not show significant differences, P, Zn and Mn, showed higher values at 0 mM and decreased as salinity increased. The K/Na ratio showed significant differences between NaCl treatments (F_4,15_ = 122.28; *p* = 0.000001) and was reduced significantly as salinity increased. The Na/K showed significant differences between NaCl concentrations (F_4,15_ = 243.55; *p* = 0.000001) and increased as NaCl concentration increased (Table 2).

## Discussion

### 4.1 Biochemical variables

Salinity causes either a decrease or an increase in the level of soluble proteins or in some cases causing them to completely absent [28]. In our case it was found that protein content decreased in chlorenchyma while it increased in the parenchyma in response to increasing salt stress (Table 1). This not unusual, since researchers have reported that in leaf tissue of different plants the soluble protein content is of high molecular weight can decrease [29] or increase [30]. Several salt-induced proteins have been identified in plants species and have been classified into two distinct groups; salt stress proteins, which accumulate only due to salt stress, and stress associated proteins, which also accumulate in response to heat, cold, drought, waterlogging, and high and low mineral nutrients.

Proline is believed to protect plant tissues against stress by acting as an osmoregulator, as well as a protectant of enzymes and cellular structure [31]. In our case, it was found that proline, in contrast to protein, increased in both chlorenchyma and parenchyma tissues in Aloe plants with increasing salinity (Table 1). These results suggest that probably proline is an important component in Aloe for osmotic adjustment during the initial stages of salinity stress. This is supported by previous studies indicating that amino-acids such as asparagine and proline play an important role in the osmotic adjustment of the plant under saline conditions [32]. In addition, [33] showed that proline in leaves of *Hibiscus tuberosus* from different areas of the plant increased with seawater irrigation. This change is probably causing leaf water potential to drop, as has been observed in other plants when subject to salinity stress [34]. In the same sense, Huang *et al*. [35] indicates that proline is induced in Jerusalem artichoke plantlets under salinity stress. More proline is accumulated in leaves as a possible protective metabolic adaptation to prevent leaf tissue from damage under salinity. Elevated proline concentrations in tissues have also been associated with drought stress. Delatorre-Herrera *et al*. [36] found that Aloe plants under water restricted regimes increased their levels of proline. According to Balibrea *et al*. [37] proline is one of the most stable amino acids, and, therefore, this amino acid probably accumulates in water-stressed Aloe plants due to its stability and low turnover. It is important to point out that although proline in the Aloe plants increased due to salt stress in this study; it was less discernible compared to other plants. For example, in water-stressed soybean plants it was found that proline increased substantially, up to 400% the initial level [38], while in our study with Aloe proline increased only 75% over the control value. The lower response of Aloe in producing proline compared to soybean may be due to the salt stress being moderate or simply differences in physiological responses.

Similarly to proline, PEP-case activity increased in both chlorenchyma and parenchyma tissues in Aloe plants with increasing salinity (Table 1). The fact that PEP-case has an important role in C4 and CAM plant metabolism suggests that this enzyme is linked to adaptation when plants experience stress conditions. Some studies have shown that PEP-case activity increased in salt treated *Sorghum bicolor* (a C4 plant), *Hordeum vulgare* (a C3 plant) and *Aleuropus litoralis* (a C3-C4 intermediate plant) [39]–[41]. The induction by salt of PEP-case activity in the facultative CAM plant *Mesembryanthemum crystallinum* has also been described by Li and Chollet [42] and by Höfner *et al*. [43] where they demonstrate that in *M. crystallinum* the induction of CAM by increased salinity involves *de novo* synthesis of PEP-case. PEP-case plays an essential role in the photosynthetic carbon metabolism of C4 and crassulacean acid metabolism (CAM) plants since it is involved in the initial fixation of atmospheric CO_2_. *Aloe vera* has the ability of great water storage capacity with efficient CO_2_ fixation by a pathway where the enzyme for CO_2_ fixation is PEP-case and not Rubisco. PEP-case has a CO_2_ affinity an order of magnitude greater than Rubisco [44]–[47]. Results in this paper show a gain in PEP-case activity in leaves from Aloe plants exposed to salinity conditions, thereby indicating a role for this enzyme and its regulatory phosphorylation in response to plants under salt stress. Although other studies have shown that increasing NaCl concentration in the growth medium significantly decreased PEP-case activity in some species such as *Zea mays* (C4 plant) and *Triticum aestivum* (C3 plant) [48], the majority of the studies have demonstrated that PEP-case enzyme and mRNA amounts increase when exposed to salt treatment [49]–[52].

### 4.2 Mineral content

Aloe has strong drought resistance and also has a certain degree of tolerance to salt stress, although it is not generally considered to be a true halophyte [15]. High accumulation of toxic salts such as Na, Cl, and B in apoplastic and cytoplasmic cell compartments of leaves can cause desiccation, cell plasmolysis and, finally, cell death due to the dramatic increase in osmotic pressure. Symptoms include chlorosis and necrosis of meristematic areas followed by necrosis of leaf margins on older and, finally, younger growth. Salt accumulation in leaves and premature leaf abscission in response to salt stress has been suggested to be a defensive mechanism used by plants to rid themselves of damaging organisms [53]. Accumulation of inorganic solutes, such as cations of Na and K and the anion Cl, can also play a role independently or in combination with other mechanisms in maintaining the osmotic imbalance caused by the salt stress and influence the osmotic potential adjustment of plant cells [54]. In the present study, the Na and Cl content of roots, stems, leaves and sprouts increased with the increase in NaCl concentration (Table 2), which are results that agree of ion content analysis in different organs (roots and leaves) of Aloe, These previously reported results indicated that Na and Cl content in tissues increased significantly, while and K and Ca transport in roots and leaves were inhibited in seedlings exposed to NaCl stress [15]. During the experimental period of the present study, Na and Cl ions accumulated in the cells of roots, stems, leaves and sprouts, showing higher Na and Cl content in sprouts followed by leaves, stems and roots (Table 2). Under NaCl stress, the addition of silicate (Si) to nutrient solution decreased significantly the Na and Cl contents in Aloe roots, stems and leaves while K content and K/Na ratio increased significantly. The addition of Si enhanced the selectivity of root to K absorption and the selectivity of stem and leaf to K translocation of Aloe under NaCl stress [19].

On the other hand, when the plants are exposed to NaCl the plant certainly absorbs a great amount of Na, which consequently causes a decrease in the contents of K [55]. In general, the majority of salt tolerant plants accumulate Na in their tissues (stems, leaves) whereas sensitive plants do not. In the present study, although NaCl stress reduced K content in roots, stems, leaves and sprouts of Aloe plants (Table 2), when the K/Na ratio was analyzed in all tissues, the average of K/Na ratio show a value of 1.11, which practically mean that for each atom of Na, a K atom is translocate to the plant tissue. ATPase provides the energy needed for uptaking K and excreting Na through the Na/H antiporter while TP-H-ATPase and TP-H-PPase provide the driving force essential for salt ion compartmentation in the vacuoles [56].

In Aloe roots under NaCl stress, Si stimulates the activities of PM-H-ATPase, TP-H-ATPase and TP-H-PPase and promotes the selectivity of K absorption and translocation and maintains ion homeostasis in cells of Aloe plants by strengthening the proton pump activity of plasma membrane and tonoplast [19]. The K/Na selectivity is an important determinant of salt tolerance and it depends on the characteristics of the transporters that mediate K and Na absorption. The ratio K/Na in the present study showed values decreasing in the following order, sprouts > leaves > stems > roots. However, mineral content of sprouts is the sum of all tissues (roots, stems and leaves). On the other hand, the fact that the sum of total Na and Cl in roots and stems of Aloe plants are very close (119.89 and 122.43 mg g^−1^ for Na, respectively; 51.17 and 52.22 mg g^−1^ for Cl, respectively) shows that translocation of Na and Cl from roots to stems in Aloe plants is high, which results in high Na and Cl content in leaves; however, the K/Na ratio is higher in leaves than in stems and roots (Table 2), which mean that the selectivity of K absorption and translocation is high, confirmed with the higher values of K than Na in leaves (Table 2). Ning *et al*. [21] found that in *Aloe vera* under salt stress, content of Na and Cl increased with the salt concentration, while an opposite trend occurred for the K and Ca content. Na was mainly accumulated in the stem, content of Na in the leaves and root was lower; Cl mainly accumulated in the leaves and the remainder of Cl content accumulated in the stem and with a lesser amount in the root. In the same sense, Zheng *et al*. [15] found that ion content in root and leaf cross sections of *Aloe vera*, the Na and Cl content increased significantly and K, Ca absorption in the roots and transport to leaves were inhibited in seedlings exposed to NaCl stress. Under high salt conditions, Blumwald *et al*. [57] have suggested that Na is able to enter the cell since the hydrated ionic radii of Na is similar to K making it difficult for the transporter to discriminate between these two ions This ion homeostasis can actually be a reflection of several different strategies that the plant uses such as diminishing the entry of Na ions into cells, extrusion of Na ions out of the cell or/and vacuolar compartmentation of Na ions. In the present study, the high accumulation of K mainly in leaves under moderate NaCl suggests a more efficient K uptake in Aloe plant. This coincides with Jacoby [58] who proposed that K accumulation represented plant adaptation to salinity because of elevated K levels act osmotically, preventing Na influx into roots and shoots. It has been suggested that not only K and Na content, but also the Na/K ratio can be used as an indicator for phytophysiological factors for screening less sensitive plant for NaCl stress [59]. The ratio Na/K in the present study had decreasing ratios in the following order, root > stems > leaves. When the Na/K ratio was considered for all tissues, an average Na/K ratio of 1.30 was found (Table 2), which can be considered to be a low ratio, meaning that Aloe plants studied can adapt to a low or moderate NaCl stress. Ning *et al*. [21] studying the effect of 100–400 mmol/L NaCl on the growth and ion distribution of two-year *Aloe vera* found similar low Na/K values and stronger transportation ability for K and Ca, which has been one of the main reasons used to explain salt-tolerance of *Aloe vera*. In the same sense, Zheng *et al.* [20] found that *Aloe vera* seedlings, showed a low transport rate of Na and Cl to leaves and suppressed uptake of K and Ca in roots during NaCl treatment. These results suggest that *A. vera* may be able to accumulate Na and Cl possibly in metabolically inactive aqueous cells in leaves and, as a result, the plant can survive and can maintain growth under saline conditions.

Other studies have also demonstrated that a high Na/K ratio indicates metabolic disorders such as a reduction in protein synthesis and enzyme activities [60], and increasing membrane permeability [61]. In addition, in the present study, the protein content in parenchyma, proline and PEP-case activity in chlorenchyma and parenchyma increased as salinity increased (Table 1). Similar to previous report with Aloe [9], in the present study cations accumulated more in roots and stems than in leaves of the Aloe plants (Table 2) which suggests that such a distribution of inorganic cations decreased the osmotic potential of the rooting environment and ensured the normal physiological function and metabolism of plant. The stimulatory effect of moderate salinity on the growth of some halophytic plants was also reported by O'Leary [62], and may be attributed to increased shoot osmotic status as a result of increased ion uptake. Reduced growth at high salinities is probably associated with reduced turgor pressure and the high energy required for massive salt secretion and osmoregulation [63]. Osmotic adjustment is an important adaptation mechanism to salinity in plants because it helps maintain turgor pressure and cell structures in stressed plants [24].

Under salt-stress conditions N uptake is limited by an accumulation of Cl and its competition with NO_3_ [61]. Nitrogen content is significantly reduced by salt stress, especially in the leaves [64]. This asseveration coincide with the results showed in the present study where N in leaves of Aloe plants decreased as salinity increased (Table 2). Similar results were reported when a low concentration of N in the leaves of *Mesembryanthemum crystallinum* and *Aloe vera* plants were found at high sodicity, which were because of less availability of N due the higher concentration of Na in the soil [1], [65]. Succulent plants of C4 photosynthesis pathway and inherently adaptive to salinity/sodicity stress require certain amount of sodium for their growth acceleration, and if such plants grow in sodium deficient soils, they are not able to use N and other nutrients effectively as desired for good productivity [66]. In the present study, although not significant differences between phosphorus content between NaCl treatments were found, this ion increased as salinity increased in Aloe stems while in other tissues not showed a defined tendency (Table 2). Other study reported increases concentration of phosphorus in the leaves of *Aloe vera* plants grown at 15 and 30 exchangeable sodium percentage (ESP) which may be interrelated to accelerated metabolic activities which promoted growth and leaf biomass. The ratio of phosphorus used in production of leaf biomass at 15–45 ESP, indicates the greatest use efficiency at 10–15 ESP levels [1]. In the present study, the decrease of calcium as salinity increased was exhibited in leaves and sprouts, while in roots and stems, a clear tendency was not observed. Salinity restricts Ca uptake and transport from roots [67], then, it would be expected that Ca contents in all tissues Aloe plant decreased under salt stress, however, it seems that Ca was uptaken from roots since Ca content was higher in leaves than stems, roots, and sprouts, however, the sum of Ca in both tissues, roots and stems, was higher than leaves (Table 2). The elevated Ca content of the control and 30 mM in leaves of Aloe may be because of less Na ion in the region of the plant roots. Similar results were found in Aloe by Rahi *et al*. [1] who reported an elevated level of Ca concentration in leaf biomass in control and at 15 exchangeable sodium percentages. Other studies have been demonstrated that low sodium stress promotes Ca absorption in plants, whereas, high level of Na disrupts the entire ionic equilibrium of soil solution around the rooting zone [68]. According with Ranganathan [69] the succulent *Aloe vera* have high calcium contents, however, Ca is considered a secondary essential macronutrient, then, it does not correlate well with growth and leaf yield of Aloe [1]. Same than those results reported previously in Aloe [1], [15], in the present study, the reduced concentration of Ca failed to alleviate toxic effects of Na concentration at greater NaCl levels, which was verify when Na/Ca ratio in roots, stems, leaves and sprouts, increased as salinity increased, being higher in leaves and sprouts at 120 mM. The lower Na/Ca ratios were found in roots and stems and no significant differences between NaCl treatments were found (data not shown). Ning *et al*. [70] found that the content of K and Ca in *Aloe vera* was lower under salt stress in contrast with the control, content of K in the leaves increased obviously and the Ca content decreased. The present study showed that magnesium decreased as salinity increased except in stems where Mg content had no obvious change (Table 2). Zan *et al*. [9] reported that in two cultivars of *Aloe vera*, Mg contents of both stem and root greatly increased, and leaf Mg content had no obvious change; in addition, the maintenance of leaf Mg observed in Aloe, contributing to the normal growth of plants against long-term salt stress. Other studies have been described that Mg deficiency accelerates the plant senescence process [71]. The influence of salinity on micronutrient concentrations in plants is highly variable. In the present study, the uptake of some micronutrients including phosphate was not impaired by exposure to NaCl in some of the tissues (Table 2). Hence some micronutrients imposed no limitation to plant growth under the experimental conditions of our study; however, the alteration of the distribution of some minerals by salt treatments of some nutrients studied, may be partly a result of a reduction in their activities caused by high concentrations Cl and Na in the nutrient solution; also, uptake may also have been reduced as a result of competition with the salt ions in the external solution.

## Conclusion

The present study reveals that *Aloe vera* has an ability to withstand salinity under moderate salt stress (0, 30, 60, 90 or 120 mM NaCl). The increases in protein and proline content and PEP-case activity as salinity increases provides some of the necessary metabolic cellular protection to the plant and influences water retention in the leaves of Aloe to reduce salt stress. In addition, the absorption and accumulation of cations in roots and stems of Aloe plants under moderate NaCl stress, up to level of 120 mM, provides the osmotic adjustment necessary for stimulating normal physiological growth, therefore Aloe can be planted in soil affected by moderate salinity and irrigated with moderate saline water such as used in the present study. Moreover, the higher K/Na ratio and lower Na/K ratio indicate that the relative salt tolerance of Aloe plants to Na is mediated by increasing K mineral uptake. This avoidance of damages increases the agronomic and physiological characteristics of Aloe plants under salt stress, making this species attractive for industrial production in arid or semiarid areas around the world associated with moderate saline soils.
